# Update of recommendations for the management of COVID-19 in patients with haematological malignancies, haematopoietic cell transplantation and CAR T therapy, from the 2022 European Conference on Infections in Leukaemia (ECIL 9)

**DOI:** 10.1038/s41375-023-01938-5

**Published:** 2023-07-17

**Authors:** Simone Cesaro, Malgorzata Mikulska, Hans H. Hirsch, Jan Styczynski, Sylvain Meylan, Catherine Cordonnier, Davide Navarro, Marie von Lilienfeld-Toal, Varun Mehra, Francesco Marchesi, Caroline Besson, Raul Cordoba Masculano, Gernot Beutel, Herman Einsele, Johan Maertens, Rafael de la Camara, ECIL 9, Marie von Lilienfeld-Toal, Rafael de la Camara, Per Ljungman, Livio Pagano

**Affiliations:** 1 Paediatric Haematology-Oncology, Department of Mother and Child, Azienda Ospedaliera Universitaria Integrata, Verona, Italy; 2 Division of Infectious Diseases, Department of Health Sciences (DISSAL), University of Genova, Genova, Italy; 3 IRCCS Ospedale Policlinico San Martino, Genova, Italy; 4 Transplantation & Clinical Virology, Department of Biomedicine, University of Basel, Basel, Switzerland; 5 Clinical Virology, Laboratory Medicine, University Hospital Basel, Basel, Switzerland; 6 Infectious Diseases and Hospital Epidemiology, University Hospital Basel, Basel, Switzerland; 7 Department of Paediatric Haematology and Oncology, Jurasz University Hospital, Nicolaus Copernicus University Torun, Collegium Medicum, Bydgoszcz, Poland; 8 Infectious Diseases Service, Internal Medicine, Lausanne University Hospital and University of Lausanne, Lausanne, Switzerland; 9 Haematology Department, Henri Mondor Hospital, APHP, and University Paris Est-Créteil, Paris, France; 10 Microbiology Service, Clinic University Hospital, INCLIVA Health Research Institute, Valencia, Spain; 11 CIBER de Enfermedades Infecciosas, Instituto de Salud Carlos III, Madrid, Spain; 12 Department of Microbiology, School of Medicine, University of Valencia, Valencia, Spain; 13 Klinik fur Innere Medizin II (Haematologie/Oncologie), Universitatsklinikum Jena, Jena, Germany; 14 Leibniz Institute for Natural Product Research and Infection Biology, Hans Knöll Institute, Jena, Germany; 15 Department of Haematology, King’s College Hospital NHS Foundation Trust, London, United Kingdom; 16 Haematology Unit, Department of Research and Clinical Oncology, IRCCS Regina Elena National Cancer Institute, Rome, Italy; 17 Service d’Hematologie Oncologie, Centre Hospitalier de Versailles, Le Chesnay, Villejuif, France; 18 Fundacion Jimenez Diaz University Hospital, Health Research Institute IIS-FJD, Madrid, Spain; 19 Department for Haematology, Haemostasis, Oncology and Stem Cell Transplantation, Hannover Medical School, Hannover, Germany; 20 Department of Internal Medicine II, University of Würzburg, Würzburg, Germany; 21 Haematology Department, University Hospitals Leuven, KU Leuven, Leuven, Belgium; 22 Department of Haematology, Hospital de la Princesa, Madrid, Spain; 23 Division of Haematology, Department of Medicine, Huddinge, Karolinska Institute, Stockholm, Sweden; 24 Department of Cellular Therapy and Allogeneic Stem Cell Transplantation, Karolinska Comprehensive Cancer Center, Karolinska University Hospital, Huddinge, Stockholm, Sweden; 25 Institute of Haematology, Faculty of Medicine and Surgery, “Sacro Cuore” Catholic University, Rome, Italy

**Keywords:** Diseases, Medical research, Haematological diseases, Epidemiology, Risk factors

## To the Editor:

In 2022, the European Conference on Infections in Leukemia, 9th edition (ECIL-9), published the guidelines for the management of severe acute respiratory syndrome infection (SARS-CoV-2) and disease (COVID-19) in hematological malignancy (HM) and haematopoietic cell transplant (HCT) patients [1]. During the year 2022, the worldwide epidemiology, morbidity, and mortality changed for the better compared to the previous two years of the pandemic due to the positive effect of public health and social measures, the mass vaccination campaigns, availability of antiviral drugs and anti-spike monoclonal antibodies (MoAbs) [2]. The achievements were partly hampered by the continuous emergence of highly contagious variants of concern, escaping the immunity acquired by infection or vaccination. Considering these significant changes, an update of the SARS-CoV-2/COVID-19 recommendations was performed at ECIL-9 Conference held in Nice, France, on 14–15 September 2022 and published on the ECIL web page (https://www.ecil-leukaemia.com/en/program). Table 1 shows the updated recommendations on prevention, diagnosis, vaccination, and therapy.

**Table 1:** Summary of ECIL-9 recommendations on prevention, diagnosis, vaccination and therapy for SARS-CoV-2/COVID-19 in adult and pediatric onco-hematological patients.

N° recommendations		Grading
Prevention		
1	Hand hygiene, face mask, distancing, and ventilation of rooms are recommended in patients with HM.	AIIu
2	Caring for a SARS-CoV-2 positive HM patient requires the use of personal protective equipment^a^ by health personnel and isolation in single room.	AIIrtu
3	Placing a SARS-CoV-2 positive HM patient into positive pressure rooms is not recommended.	AIII
5	Ensure the best possible treatment of underlying HM disease weighing individual patient related risks and benefits.	AIIu
6 New	*In HM patients with asymptomatic SARS-CoV-2 infection (and no previous COVID-19), defer chemotherapy, HCT, CAR-T therapy, therapy with monoclonal antibodies or other targeted therapies after assessment of clinical risk/benefit ratio*	*BIIu*
7 New	*In HM patients with COVID-19, defer chemotherapy after assessment of clinical risk/benefit ratio*	*BIIu*
8	In HM patients with COVID-19, defer cellular therapy (HCT, CAR-T).	AIIt
9 New	*In case of patients persistently shedding the virus after complete recovery from COVID-19, defer chemotherapy, HCT, CAR-T therapy, therapy with monoclonal antibodies, and other targeted therapies after assessment of the clinical risk/benefit ratio*	*BIIu*
10 ChW	Therapy with JAK2-inhibitors and TKI/BTKi should be continued in HM patients with SARS-CoV-2 infection.	AIII
11	In case of high circulation of SARS-CoV-2 virus, adapt the treatment plan to reduce the visits to the hospital and the risk of SARS-CoV-2 exposure, i.e.	
	using telemedicine,	AIII
	using erythropoietic growth factors	BIII
12	Avoid using granulocyte-colony stimulating factor outside of the recommended indications because of the risk of worse COVID-19 outcomes.	BII
Diagnosis		
13 ChW	NAT assays are recommended for the diagnosis of SARS-CoV-2 infection inside and outside of hospitals.	AII
14	SARS-CoV-2 molecular NAT assays should target at least two distinct viral gene sequences.	AIIt
15 New	*The performance of SARS-CoV-2 molecular NAT assays should be evaluated for newly emerging variants*.	*AIIt*
16	Rapid antigen testing validated for circulating variants should be reserved for rapid point-of-care diagnosis.	AII
17	Rapid antigen testing validated for circulating variants should be confirmed by molecular NAT assays.	AII
18 New	*Testing for SARS-CoV-2 RNA in saliva or oropharyngeal gargle may be considered for symptomatic HM and HCT patients*.	*BIIt*
19 New	*Testing for SARS-CoV-2 RNA in saliva or oropharyngeal gargle may have a lower sensitivity in asymptomatic HM and HCT patients, but may be considered for serial (repeated) screening*.	*BIII*
20	Clinical virology laboratories need to document proficiency in external SARS-CoV-2 quality accredited programs.	AII
21	Nasopharyngeal or combined naso-oropharyngeal swab (with nostrils and throat with one swab) are recommended to diagnose SARS-CoV-2 upper respiratory tract infections.	AII
22	Low respiratory tract fluid sampling (tracheal aspirate, bronchoalveolar lavage) for SARS-CoV-2 is not recommended in HM and HCT patients with positive nasopharyngeal or naso-oropharyngeal swab molecular test, unless there are clinical indications for viral, bacterial, fungal, or parasitic infections in the lower respiratory tract.	AII
23 ChW	Lower respiratory tract fluid (tracheal aspirate, bronchoalveolar lavage) is recommended to diagnose SARS-CoV2 LRTI in HM and HCT patients with negative nasopharyngeal-naso-oropharyngeal molecular test.	AII
24	In symptomatic HM and HCT patients with symptoms/signs of LRTI and negative SARS-CoV-2 molecular tests, diagnostic testing should be expanded to other pathogens.	AI
27 ChW	Testing for SARS-CoV-2 RNA or antigens in blood is not recommended for diagnosis or management of COVID-19.	AI
28 ChW	Performing serial (semi-quantitative) NAT is recommended to inform decisions regarding infection control or deferral of therapy or HCT.	BIII
29 New	*The detection of SARS-CoV-2-RNA or N-protein in blood correlates with a more severe course of COVID-19, but harnessing this information for clinical management requires further study*.	*Not graded*
30 New	*The role of qualitative SARS-CoV-2 E-gene sgRNA for monitoring the viral response of HM or HCT patients treated with antivirals requires further study*.	*Not graded*
31	SARS-CoV-2 genome quantification of Ct-values > 30 is associated with low/absent risk of transmission provided adequate sampling quality.	BII
32	SARS-CoV-2 antibody assays are not recommended to diagnose a new-onset acute SARS-CoV-2 infection /COVID-19.	AII
33	Immunocompromised persons such as HM and HCT patients may have mitigated antibody responses.	BII
34 New	*The role of quantitative antibody assays calibrated to the 1*^*st*^ *WHO-approved SARS-CoV-2 antibody standard is not defined for routine clinical-decision making regarding administration of booster vaccine doses or monoclonal antibody therapies*.	*CIII*
35	Antibodies assay targeting N-protein can be considered to ascertain previous SARS-CoV-2 exposure.	AII
36	Antibodies assay targeting S-protein can be considered to ascertain vaccine response or previous exposure to SARS-CoV-2.	AII
37 New	*Antibody assay targeting to N-protein can be considered in patients with suspected multi-inflammatory syndrome in children (MIS-C)*.	*AII*
38 New	*The use of “in house” or commercially-available T-cell assays for the diagnosis or the management of SARS-CoV-2 infection requires further study*.	*Not graded*
Vaccination General recommendations for all HM patients including HCT or CAR-T cell recipients		
39 New	*Except in specific conditions where the expected response rate is very low, HM patients should receive a three-dose program of mRNA vaccines or a two-dose program with protein subunit vaccine according to recommendations by international and national authorities and authorized age range, starting preferably before treatment of the underlying disease or HCT, or during maintenance or off-therapy phase.*	*AIIt/u*
40 New	*The interval between the first two doses should be at least 3 weeks and the interval between the 2nd and 3rd dose mRNA vaccine should be one, but preferably 3 months.*	*BIIt/u*
41 New	*Additional (booster) dose(s) of mRNA vaccine should be considered after at least 3 months from the 3rd dose.*	*BIIt/u*
42 New	*For patients having COVID-19 infection, booster dose(s) should be delayed to at least 3 and preferably 4 months after the COVID-19 episode.*	*Not graded*
43 ChW	Whichever vaccine response, HM patients should be informed of the ongoing risk of COVID-19 despite vaccination and follow the hygiene and social distancing recommendations of their community or country.	BIIt
44	The vaccination of the house-hold contacts of HM patients, including children, and according to the EMA approval for specific age groups, is recommended.	AIIth
45 ChW	The vaccination of HM patients with previous SARS-CoV-2/COVID-19, according to the indications of international and national authorities, is recommended	AIItu
46 New	*Prophylaxis with MoAbs should not prevent vaccination against COVID-19 in situations where such are indicated.*	*BIII*
Specific guidelines for non-transplanted HM patients		
47 New	*There is until now no specific safety issue of COVID-19 vaccination with mRNA vaccines in non-transplanted HM patients. COVID-19 vaccination should not delay the treatment of the underlying disease.*	*Not graded*
48 New	*Uncontrolled data and one meta-analysis indicate better responses with the mRNA1273 over the BNT162b2 vaccine. However, no evidence-based recommendation can be given on choice of vaccine in the absence of prospective comparative trials. The choice of the vaccine should be in accordance with official EMA recommendations and country recommendations.*	*Not graded*
49 ChW	Patients who have been vaccinated before or during hematological treatment should be assessed 3–6 months after the end of treatment and revaccinated if they have low antibody titers.	BIII
50	Considering the low rate and heterogeneity of the response in some HM and therapies, vaccinated patients should be assessed for their antibody response 3–5 weeks after the last dose.	BIIu
Specific guidelines for patients with LPD or AL		
51 New	*Patients with an expected low or very low response rate to vaccine (eg. anti-CD20 MoAb therapy ongoing or within the 6–12 months from the last dose, BCMA targeted bispecific monoclonal antibody (Belantamab-mafodotin) therapy, induction chemotherapy for AL, profound hypogammaglobulinemia (<4 g/L), deep lymphopenia (<500/μL), do still benefit from vaccination.*	*BIIu*
52 New	*However, these patients should undergo testing for anti-S antibodies one month after each vaccine dose (from dose 2) in order to assess their response, and discuss the use of pre-exposure MoAbs or other preventative measures.*	*Not graded*
Specific recommendations for HCT recipients		
53 ChW	HCT recipients should receive COVID-19 vaccine with a three-dose primary schedule of mRNA vaccine.	AIIut
54	Vaccination should preferably be initiated at least 6 months after HCT if transmission of SARS-CoV-2 in the community is low.	BIIu
55 New	*Earlier vaccination should be considered if there is high prevalence of SARS-CoV-2 in the community. However, early vaccination is associated with a lower likelihood for an immune response.*	*BIIu*
56	There is a risk for worsening/eliciting GVHD in allogeneic HCT recipients. This risk needs to be considered when deciding about time for vaccination.	AIIu
57 New	*It is possible that the risk for GVHD using the protein-subunit vaccine might be lower and could be considered in individual patients after careful risk assessment.*	*Not graded*
58 New	*Additional doses are able to improve the immune response both by allowing an increased proportion of patients to seroconvert and to increase the antibody levels*. *It is therefore recommended that patients should*: *a) receive booster doses,* *b) preferably with the new updated bivalent vaccines (according to authorizations for age)*.	*AIIt* *BIIt*
59	Based on data from other vaccines, it is likely that immunity obtained from either pre-transplant SARS-CoV-2 infection or vaccination will be wiped out by the transplant procedure. However, no data currently exists regarding this issue. However, it seems logical from a risk/benefit assessment that such patients should have a full dose new vaccine schedule after transplantation.	BIII
60 New	*Such repetition of a complete vaccine schedule will over time result in a large number of vaccine doses and the safety profile of such an approach is currently unknown.*	*Not graded*
61 ChW	HCT patients with previous COVID-19 should be vaccinated with a full program.	AIItu
Specific recommendations for HCT donors		
62	There is no specific recommendation for vaccinating stem cell donors for any other purpose than protecting the donor. However, previous vaccination of the donor might reduce the risk to jeopardize the donation.	Not graded
63 New	*There have been reports of transfer of donor immunity to allogeneic HCT recipients. However, whether this can result in protection against SARS-CoV-2 infection or disease in the recipient is unknown.*	*Not graded*
Specific recommendations for patients treated with CAR-T cells		
64 New	*Patients with B-cell aplasia after treatment with CD19+ CAR T cells are very unlikely to mount antibody responses but repeated vaccine doses might show some benefit*. *After vaccination, T cell responses can be elicited in a majority of patients, but the importance for protection in patients is currently unknown.*	*Not graded*
65 New	*Timing of vaccination should be based on individual consideration taking into consideration the immune status of the patient.*	*BIII*
66 New	*Patients with B-cell aplasia after treatment with CD19+ CAR T cells should receive pre-exposure monoclonal antibody prophylaxis.*	*AIIt*
Therapy		
67 ChW	In moderately or severely immunocompromised HM patients, pre-exposure prophylaxis is recommended with long-acting anti-SARS-CoV-2 monoclonal antibodies if active against circulating variants, irrespective of previous vaccination.	BIIt
68 ChW	In HM patients at high risk for COVID-19 progression (not vaccinated, vaccine non-responders or not expected to respond to vaccine) post-exposure prophylaxis is recommended with anti-SARS-CoV-2 monoclonal antibodies if active against the circulating variants.	AIIt
69 ChW	In hematological patients with mild-moderate COVID-19, early treatment is recommended, with the followings:	AI
	a) anti-SARS-CoV-2 monoclonal antibodies, if active against the circulating variants	AIIt
	b) nirmatrelvir/ritonavir	AIIt
	c) remdesivir *(main drawback: intravenous administration)*	BIIt
	d) molnupiravir *(main drawback: lower efficacy)*	BIIt
	Dexamethasone should not be used in early treatment of mild-moderate COVID-19	DIIt
70 ChW	In HM patients with moderate COVID-19 requiring oxygen support, or severe COVID-19 (saturation <90–94%%, respiratory rate >30/min) the following treatments are recommended:	
	a) Dexamethasone	AIIt
	b) Remdesivir	BII t
	c) If the patient is seronegative:	
	- monoclonal antibodies, if active against the circulating variants or	BIIt
	- high titer convalescent plasma^b^, if MoAbs are not available	CIII
	d) If severe COVID-19 inflammation^c^, including worsening despite dexamethasone, add the second immunosuppressant:	AIIt
	- anti-IL-6 (tocilizumab, sarilumab) or	BIIt
	- JAK –inhibitor (baracitinib/tofacinib^d^)	CIIt
	- anti-IL1 (anakinra)^e^	CIIt
71 ChW	In patients with critical COVID-19 (ARDS, sepsis, septic shock, MIV, NIV or vasopressor therapy, the following treatments are recommended:	
	a) dexamethasone	AIIt
	b) remdesivir	CIIt
	c) monoclonal antibodies if active against the circulating variants in NIV patients (no data in MIV patients).	CIIt
	Add 2^nd^ immunosuppressant, if COVID-19-related inflammation is present^c^, as:	AIIt
	- Anti-IL-6 (tocilizumab, sarilumab)	BIIt
72 New	*In the most severely immunocompromised HM patients, the proposed treatment strategies for challenging situations are:* *1. For the new asymptomatic infection, follow the same recommendations of mild COVID-19 to reduce the risk of progression, the length of shedding and the risk of delaying chemotherapy/transplant*. *2. For a clinical or virological rebound (defined as reappearance of symptoms and/or SARS-CoV-2 positivity shortly after clinical improvement), consider a new course of treatment since all the treatment schedules with antivirals are short.* *3. For prolonged COVID-19 or prolonged asymptomatic SARS-COV-2 infection:* *• Consider treatment with a combination of antivirals or combination of antiviral(s) and MoAbs (or high titer CVP) in order to obtain clinical improvement and prevent disease progression*. *• Consider potential development of resistance to MoAbs, and, less frequently, to antivirals (in particular remdesivir).*	*BIII*

*ChW* change of wording, *ARDS* Acute respiratory distress syndrome, *ECIL* European Conference on Infections in Leukemia, *HM* hematological malignancy, *SARS-CoV-2* severe acute respiratory syndrome Coronavirus-2, *COVID-19* Coronavirus disease 2019, *HCT* haematopoietic cell transplantation, *CAR-T* chimeric antigen receptor T, *NAT* nucleic acid test, *LRTI* lower respiratory tract infection, *Ct* cycle threshold, *EMA* European Medicine Agency, *BCMA* B cell maturation antigen, *LPD* lymphoproliferative disease, *AL* acute leukemia, *MoAbs* (anti-spike) monoclonal antibodies, *MIV* mechanical invasive ventilation, *NIV* non-invasive ventilation, *TKI* Tyrosine Kinase inhibitor, *BTKi* Bruton Tyrosine Kinase inhibitor.

^a^N95/FFP2 facial mask, gloves, glasses, visor, gown.

^b^As per FDA definition.

^c^E.g. CRP > 75 mg/dl in the absence of bacterial coinfection (based on RECOVERY trial) or other available inflammation parameters or scores (if not altered due to the underlying hematological disease).

^d^Baricitinib to be preferred, tofacitinib only if other options are not available. Note that the effects of immunomodulatory therapies targeting COVID-19 on the course of disease in already immunosuppressed patients are poorly understood and deserve special consideration.

^e^Patients with a plasma soluble urokinase plasminogen activator receptor (suPAR) level ≥ 6 ng/mL or identified in a scoring system as likely to have high suPAR levels.

In the prevention part, the added recommendations highlight the importance to personalize the decision to defer chemotherapy, HCT, chimeric-antigen-receptor-T (CAR-T) therapy, and other non-cellular therapies in asymptomatic patients or in patients with prolonged viral shedding, based on the individual risk/benefit ratio assessment. The deferral of treatment to avoid the risk of progression to severe COVID-19 is no longer considered mandatory but should weigh the urgency of the patient’s treatment, the effect of postponing therapy on the risk of relapse or progression of the underlying disease, the degree of immunocompromise, the remission status against the possibility to control viral replication by antivirals and/or MoAbs.

In the diagnostic part, the new recommendations highlight the need for the surveillance and external quality assurance of the performance of nucleic acid tests and rapid antigen assays on newly emerging (sub)variants. While nucleic acid tests on nasopharyngeal specimen remain the reference, the use of less invasive samples such as oropharyngeal gargle or saliva can be a reliable alternative only for the symptomatic patient. Sampling of lower respiratory tract fluids is not needed for patients testing positive for SARS-CoV-2 RNA on upper respiratory tract specimens, but is recommended in case of a differential diagnosis with other clinical etiologies, of suspicion of coinfections (together with the appropriate diagnostic workup with blood and sputum cultures, fungal serum markers, urine antigens for pneumococci and Legionella) or if upper respiratory tract sampling is negative but SARS-CoV-2 is suspected. The prognostic significance of the qualitative and quantitative detection of SARS-CoV-2 RNAemia or N-antigenemia requires further investigation [3]. Testing for SARS-CoV-2 spike antibodies, whose levels correlate with serum neutralizing activity, is not recommended for clinical decision-making regarding vaccine boosters and administration of MoAbs [4]. Despite T-cell responses having a protective role after natural infection and vaccination, the use of SARS-CoV-2 T-cell assays is not recommended for clinical decision-making [5].

In the vaccine part, the recommended mRNA vaccine schedule for the vulnerable population of HM and HCT patients is adjourned to the three-dose primary schedule followed by an additional vaccine dose.

A third dose improved the rate of seroconversion in patients who had not responded to two doses of mRNA vaccine [6, 7] and a 4th dose was safe and could increase antibody titers [8]. It is highlighted that there are no safety issues in non-transplanted HM patients and that vaccination should not delay the treatment of the underlying disease. With the spread of the Omicron variant many patients with HMs and after HCT have now developed SARS-CoV-2 infections both before and after having received vaccinations. It is recommended that the interval between COVID-19 and subsequent boosters should be at least three and preferably four months. An important change in the recommendations is that also patients with expected poor or very poor responses, such as those receiving therapy with anti-CD20 antibodies or being 6–12 months after the last dose, those with profound hypogammaglobulinemia (<4 g/L), deep lymphopenia (<500/μL), receiving B-cell-maturation-antigen (BCMA) targeted bispecific therapy and patients starting induction chemotherapy for acute leukemia, might still benefit from vaccination also by inducing a T-cell response.

Regarding patients after allogeneic HCT, several studies have shown that additional doses of vaccine can result in seroconversion of patients not having responded to the first two or three doses [9]. At the time of the meeting, the mRNA adapted bivalent vaccines targeting original strains and Omicron subvariants BA.4/5 had just been licensed, initially as booster dose and also for primary immunization in the following months. The of use of updated bivalent vaccines was supported in the guidelines even though it was recognized that there was no information regarding their efficacy in severely immunocompromised patients

Although it is not a new recommendation, increasing evidence exists regarding the risk for the development or worsening of graft-versus-host disease after mRNA vaccination and this risk should be considered when planning vaccination schedule. It was noted that the transfer of donor-specific SARS-CoV-2 immunity has been documented but it is unknown if such transfer can be protective early after HCT or improve the vaccine response of the recipient.

A new section of the recommendations deals specifically with patients having received CAR-T cell therapy. Various studies have shown that patients with B-cell aplasia are very unlikely to have an antibody response but T-cell responses can be seen in a majority of them [10]. As in other populations, the protective value of these responses remains, however, unknown. Also in CAR-T recipients additional doses resulted in improved vaccine responses.

In the therapy part, the main changes concerned the loss or significant reduction of activity of all authorized MoAbs against the new VOCs, and the increased availability of three antivirals for early treatment of mild/moderate COVID-19 [11]. MoAbs had been previously shown effective in the prevention and treatment of mild/moderate COVID-19, and, in seronegative patients, of severe COVID-19. Their limitations included the need for intravenous administration and the risk of developing in-vivo resistance in immunocompromised patients due to prolonged viral shedding and ineffective viral clearance resulting from the lack of T-cell immunity. For pre-exposure prophylaxis with long-lasting antibodies, the increase of dose has been proposed to counteract the loss of efficacy against the BA.1 Omicron variant in February 2022, but even higher doses are currently not expected to be effective. Therefore, the recommendation is to use (probably new ones) MoAbs for prophylaxis and treatment in the HM population only if they are active against circulating VOCs.

Antivirals are the cornerstone of therapy since their activity is not influenced by VOCs, and randomized trials demonstrated that, when administered within 5 or 7 days from symptom onset, they were effective in reducing the rate of hospitalization or death in unvaccinated outpatients with mild/moderate COVID-19 who had risk factors for severe COVID-19 [12–14]. Although few HM patients were included, this population is expected to gain maximum benefit from anti-viral treatment, as demonstrated in observational studies [15].

Oral nirmatrelvir/ritonavir or intravenous remdesivir are the first choices based on efficacy data. Drug-drug interactions limit the use of nirmatrelvir/ritonavir, but the careful reduction of immunosuppression or targeted therapy agents can allow its use in most HM patients. Molnupiravir use is limited by the lower efficacy in the randomized trial (relative risk reduction of 30%, compared to 87% of nirmatrelvir/ritonavir or remdesivir), the lack of European Medicine Agency (but not FDA) authorization and, therefore, the unavailability in some countries. The advantages of molnupiravir include absence of drug-drug interactions and the possibility to use in case of renal failure (ClCr <30 ml/min).

The data available for high titer of convalescent plasma (CVP) does not support its role in the routine treatment of mild/moderate COVID-19. Considering the polyclonal protection given by CVP, less influenced by protein-S mutations which led to the loss of activity of MoAbs, CVP might be useful in immunocompromised patients, in addition to antivirals.

In severe or critical COVID-19 in HM, remdesivir treatment is recommended together with steroid treatment and a second immunosuppressant (mainly an anti-IL-6 agent), if required. In the HM patients, when choosing the second immunomodulator, the recent hematological treatment should be also considered, especially if patients are already on ruxolitinib, which should not be discontinued in case of SARS-CoV-2 infection. No data suggest the need for different treatment in HM children compared to HM adults, although children have a much lower risk of severe COVID-19 and the data are more limited. The reason for providing early treatment to children could be hastening the cure from SARS-CoV-2 infection to allow continuing HM treatment program.

In conclusion, the ECIL9 2022 provided the updated set of recommendations for the management of COVID-19 in HM and HCT patients. The advancements in knowledge on SARS-CoV-2/COVID-19 and massive economical investments in immunization and treatment led to improvement of morbidity and mortality figures although the pandemic is not declared over. Many areas of research are still open and include the role of a combination of antivirals and MoAbs, the evaluation of prolonged courses of antivirals, the management of the underlying disease/deferral of transplant in case of positivity, the role of recent high titer CVP, the use of T-cell therapies against SARS-CoV-2, and the risk of complications during HCT in patients with previous COVID-19.
